# Enhancing spinal bone anchor pull-out resistance with an L-shaped anchor

**DOI:** 10.1371/journal.pone.0302996

**Published:** 2024-05-08

**Authors:** Esther Paula de Kater, Michiel Norbert Blom, Teunis Cornelis van Doorn, Quoc Huy Tieu, David Justin Jager, Aimée Sakes, Paul Breedveld

**Affiliations:** 1 Department of BioMechanical Engineering, Bio-Inspired Technology Group, Faculty of Mechanical Engineering, Delft University of Technology, Delft, Netherlands; 2 Department of Electronic and Mechanical Support Division, Faculty of Electrical Engineering, Mathematics and Computer Science, Delft University of Technology, Delft, Netherlands; National Trauma Research Institute, AUSTRALIA

## Abstract

The success rate of spinal fusion surgery is mainly determined by the fixation strength of the spinal bone anchors. This study explores the use of an L-shaped spinal bone anchor that is intended to establish a macro-shape lock with the posterior cortical layer of the vertebral body, thereby increasing the pull-out resistance of the anchor. The performance of this L-shaped anchor was evaluated in lumbar vertebra phantoms (L1-L5) across four distinct perpendicular orientations (lateral, medial, superior, and inferior). During the pull-out experiments, the pull-out force, and the displacement of the anchor with respect to the vertebra was measured which allowed the determination of the maximal pull-out force (mean: 123 N ± 25 N) and the initial pull-out force, the initial force required to start motion of the anchor (mean: 23 N ± 16 N). Notably, the maximum pull-out force was observed when the anchor engaged the cortical bone layer. The results demonstrate the potential benefits of utilising a spinal bone anchor featuring a macro-shape lock with the cortical bone layer to increase the pull-out force. Combining the macro shape-lock fixation method with the conventional pedicle screw shows the potential to significantly enhance the fixation strength of spinal bone anchors.

## Introduction

### Spinal fusion surgery

**Fig 1:**
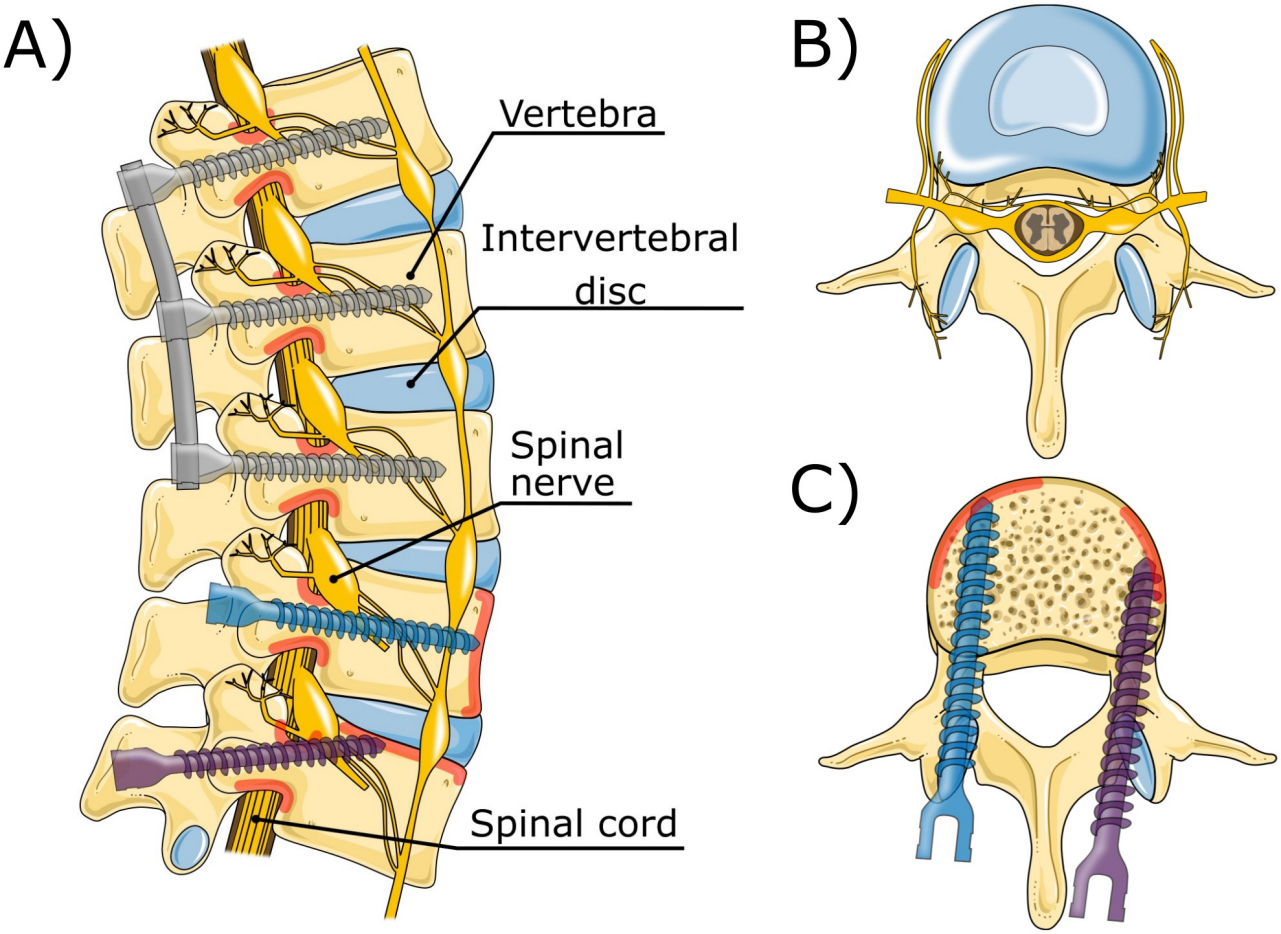
Lumbar vertebrae anatomy and placement of pedicle screws (grey), bi-cortical pedicle screws (blue), and cortical pedicle screws (purple). The contact area with the cortical layer using such screws is indicated in red. A) Lumbar vertebrae (L1-L5), intervertebral discs, spinal cord nerves and pedicle screw placement (sagittal plane). B) Lumbar vertebra (transverse plane). C) pedicle screw placement (transverse plane). Illustration adapted from Servier Medical Art by Servier, licensed under a Creative Commons Attribution 3.0 Unported License.

Spinal fusion surgery is an orthopaedic procedure that aims to fuse adjacent vertebrae to enhance spinal stability, alleviate pain, and address spinal deformities [1]. The gold standard to achieve spinal fusion entails the use of rods, firmly secured to the vertebrae with pedicle screws inserted through the pedicles into the vertebral body (Fig 1A). The overall success of spinal fusion surgery is highly dependent on the fixation strength of these pedicle screws within the vertebra, as even the slightest micro-movement between the adjacent vertebrae can hamper the desired fusion [2].

Although pull-out of pedicle screws is a very unlikely cause of implant failure, the fixation strength of pedicle screws is commonly quantified through the measurement of pull-out force, which represents the axial force required to pull the screw from the vertebra [3]. This pull-out force is the result of the interaction of the pedicle screw with the vertebra. Vertebrae comprise a thin compact layer of cortical bone that encapsulates the porous cancellous bone. The cortical bone layer shows considerable resilience against external forces due to its dense structure. While cortical bone possesses the potential to offer great fixation strength for spinal bone anchors, the majority of the currently used pedicle screws are embedded within the porous cancellous bone. Only the section of the screw located within the pedicle is in contact with the cortical bone layer. Remarkably, this relatively small section is responsible for 60% of the overall pull-out resistance [4, 5].

Correct placement of the pedicle screw is vital for the success of the spinal fusion surgery. The spinal column comprises vertebrae separated by intervertebral disks providing both stability and mobility to the spine. Additionally, the vertebrae play an important role in protecting the spinal cord, which runs through the spinal canal (Fig 1A and 1B). The spinal column is surrounded by delicate and vital structures such as the spinal cord, vascular and nervous tissue. This presents a challenges in the placement of pedicle screws, as a misalignment may result in damage to these critical anatomical structures or lead to suboptimal screw fixation [6, 7]. When striving to enhance the fixation strength, a larger screw diameter appears advantageous, as it increases contact with the cortical bone layer within the pedicle, consequently increasing the fixation strength [8]. However, a larger screw diameter also elevates the risk of breaching the cortical bone layer and potentially damaging the surrounding anatomy.

### Anchor trajectory optimisation

The search for enhanced pedicle screw fixation has led to a variety of innovative strategies, including various screw thread types and the use of cement-augmented screws [9, 10]. Another approach to enhance the fixation strength of spinal bone anchors entails optimising the screw trajectory. For instance, in bi-cortical placement the pedicle screw is inserted to ensure fixation of screw tip within the anterior cortex [2, 11] (Fig 1A and 1C). This screw trajectory offers increased fixation strength, even with an average cortical bone layer thickness of only 0.4 mm [2, 11, 12]. Another alternative screw trajectory is the cortical bone trajectory for lumbar pedicle screw placement. This more lateral and caudo-cranial trajectory engages more cortical bone, thereby enhancing the pull-out resistance by approximately 30% [13, 14] (Fig 1A and 1C).

The posterior cortex of the vertebral body, with its perpendicular orientation to the pedicle, presents an ideal opportunity for a spinal bone anchor to establish a macro-shape lock, potentially enhancing pull-out resistance. However, the posterior cortex is currently underutilised in terms of contributing to the fixation strength of pedicle screws due to design and placement limitations that do not accommodate the required L-shape. A number of patents feature spinal bone anchors with outward-curving sections aimed at creating an L-shape macro shape-lock with the posterior cortex of the vertebral body [15, 16]. However, to our knowledge, these designs have not undergone testing in a close to clinical setting. Furthermore, there remains a limited understanding of the potential fixation strength of a macro-shape lock with the posterior cortex of the vertebral body and the optimal orientation for these types of L-shaped anchors. The anchor design presented by Shae *et al*. [17] demonstrates the potential of an expanding lateral pin, employing a rotational motion for anchor deployment. However, the undesirable consequence of this rotational motion of the lateral pin is the compression of the surrounding cancellous bone, potentially compromising the fixation strength of the anchor.

### Goal of this study

The goal of this research is to investigate and optimise the use of L-shaped spinal bone anchor designs that leverage the advantages of a macro-shape lock with the posterior cortex of the vertebral body to enhance pull-out resistance. This study aims to assess the fixation strength and potential benefits of these anchor designs, including their optimal orientation. Additionally, we seek to expand our understanding of the safety implications associated with these anchor designs, particularly in unforeseen circumstances that could lead to complete anchor pull-out from the vertebra. Our research aims to contribute valuable insights into improving the effectiveness and safety of spinal fusion procedures by enhancing the fixation strength of spinal bone anchors.

## Method

### Anchor design

**Fig 2:**
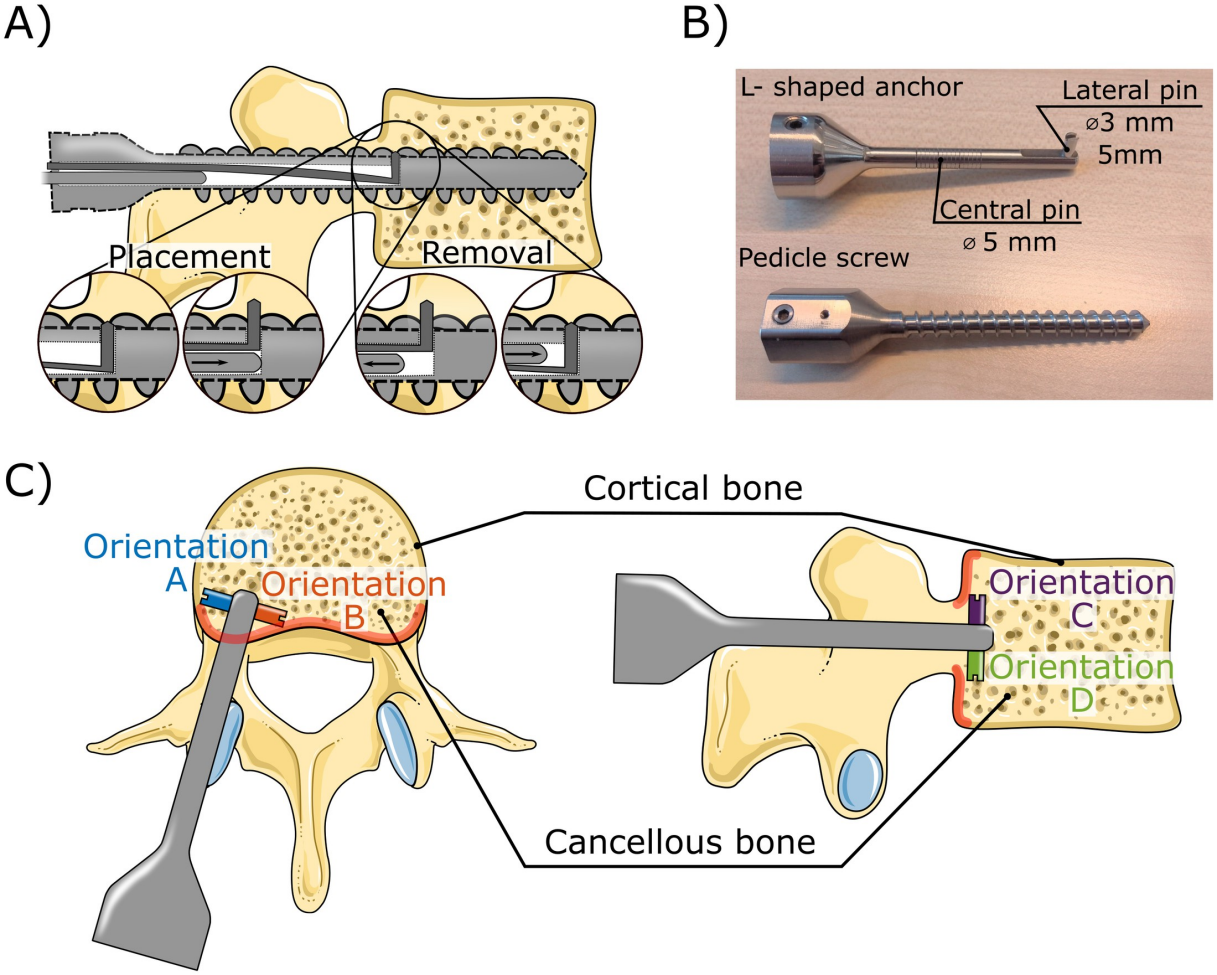
L-shaped spinal bone anchor design and placement. A) Possible expansion mechanism that allows placement of the L-shaped anchor through a single entry point as well as the removal of the anchor. B) Photograph of the L-shaped spinal bone anchor without screw thread, and a reference pedicle screw. The L-shape anchor comprises a central pin with a series of grooves that serve as indication of the insertion depth. C) Four orientations (A-D) of the L-shaped anchor that were evaluated in the experiment. The proximal cortex of the vertebral body is indicated in red. Illustration adapted from Servier Medical Art by Servier licensed under a Creative Commons Attribution 3.0 unported license.

The currently utilised pedicle screws are inserted through the pedicle of the vertebra into the vertebral body to provide essential stability and fixation strength [4, 5]. Introducing a pedicle screw with a lateral pin resulting in an L-shaped anchor through the same pedicular path allows for the same level of fixation strength as the currently used pedicle screw with the added possibility to establish a robust macro-shape lock with the posterior cortex of the vertebral body, thus enhancing the pull-out resistance. Fig 2A presents our conceptual design of a pedicle screw with an integrated L-shaped rod designed to create the desired macro-shape lock with the posterior cortex. The expansion of the rod enables placement of the pedicle screw through a single entry hole, similar to current pedicle screws. Furthermore, the expansion of the lateral pin is achieved by a translational motion which does not result in the undesired compression of the surrounding cancellous bone as a rotational expansion would. Besides placement, removal of spinal bone anchors is of importance, as implant removal can be required due to non-fusion, occurrence of infection or implant loosening [18]. The lateral pin of the L-shaped anchor can be retracted by removing the expansion rod, and subsequently introducing it such that it will result in retraction of the pin as illustrated in Fig 2A.

The primary research objective of this study is to investigate the potential additional fixation strength of this macro-shape lock compared to the conventional pedicle screw, and determine the optimal orientation of the L-shaped anchor for both strength and safety. To achieve this, an L-shaped anchor without screw thread comprising a central pin and a lateral pin at the tip has been developed (Fig 2B). This anchor is not expandable and is merely intended as a means of researching the potential of utilising a macro-shape lock with the cortical bone layer. The length of the lateral pin of the L-shaped anchor was designed to be equal to the diameter of the anchor as this is theoretically the maximum length that would allow for the anchor to be placed through a single entry hole following the design presented in Fig 2a. In this research four orientations of the L-shaped anchor each with a 90-degree rotation were considered (A-D, Fig 2C).

### Experimental goal

The primary aim of this research is to explore the effect of an L-shaped macro lock with the proximal cortical bone layer on the fixation strength of spinal bone anchors. An experiment was carried out to assess the influence of the anchor orientation across various lumbar vertebra. The two most important factors in evaluating spinal bone anchors are the fixation strength and safety. These two factors were investigated based on 1) pull-out resistance of the anchor and 2) damage to the vertebra after complete pull-out of the anchor. Both factors were compared to the conventional pedicle screw.

### Experimental variables

The following independent variables were varied during the experiment:

- **Anchor orientation**: The pull-out experiment was conducted using the L-shaped anchor in four orientations with the lateral pin pointing in the cranial direction, caudal direction, medial direction, and lateral direction, respectively (Orientation A-D, Fig 2C).
- **Vertebra type**: The pull-out experiment was conducted using lumbar vertebra phantoms provided by Synbone^®^ (Spine Vertebra L1-L5, LSS material). These vertebra phantoms closely mimic real vertebrae, featuring a porous cancellous bone structure [19].
- **Anchor type:** The pull-out experiment was conducted with the L-shaped anchor as well as with a conventional pedicle screw as presented in Fig 2B. The pedicle screw was only tested in a single orientation and only in the L2 vertebra.

The following variable was kept constant during the experiment:

- **Pull-out velocity**: The L-shaped anchor was pulled out of the vertebra at a constant velocity of 0.5 mm/s.

The following dependent variables were measured during the experiment:

- **Pull-out force**: The pull-out force of the anchor was measured continuously during the experiment. This allows for the determination of the maximum pull-out force (i.e., the maximum force required to pull the anchor from the vertebra) and the initial pull-out force (i.e., the force required to initiate the pull-out of the anchor from the vertebra).
- **Pull-out distance:** The relative displacement of the anchor with respect to the vertebra was measured. This measurement, combined with the continuously measured pull-out force allows for the determination of the initial pull-out force.
- **Pull-out damage**: The damage to the vertebra after complete pull-out of the anchor was categorised as follows: 1) clean pull-out: no breach detected, 2) posterior breach: breach at the entry point of the anchor, 3) pedicle breach: breach of the cortical layer of the pedicle. Damage to the cortical bone layer could indicate damage to the surrounding anatomy and is considered less safe.

### Vertebra preparation

**Fig 3:**
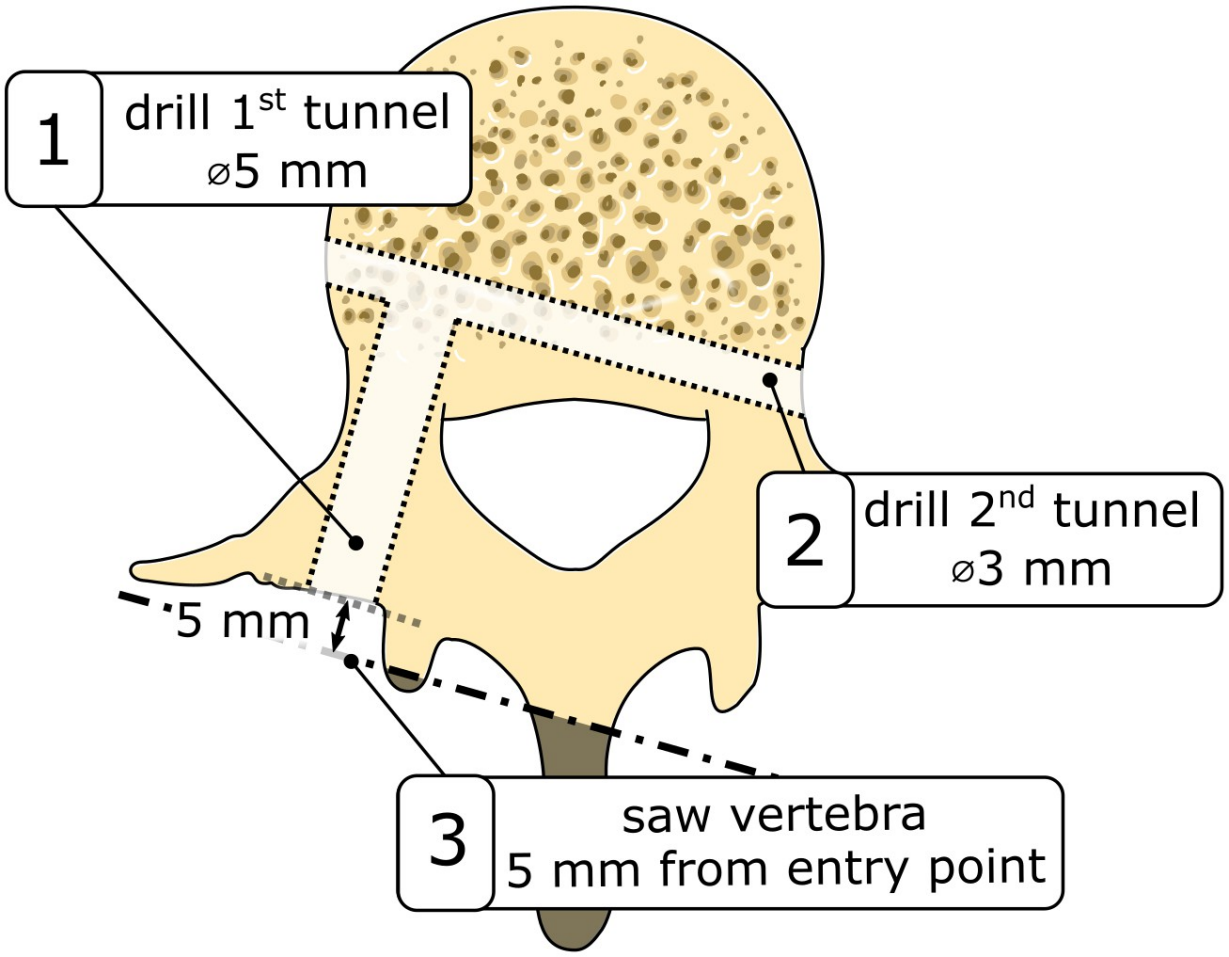
Vertebra preparation steps before placement of the L-shaped anchor.

To accommodate the central pin of the L-shaped bone anchor, a first ∅5mm tunnel was drilled through the central axis of the pedicle of the vertebra phantom taking the variations in angulation across different spinal levels (L1-L5) into account (Fig 3, step 1). Subsequently, a perpendicular ∅3mm tunnel was drilled to accommodate the lateral pin of the bone anchor (Fig 3, step 2). For this, a 3D printed guide was utilized to guarantee both the perpendicularity of the two tunnels and their correct alignment within the vertebral anatomy. The same procedure for drilling the first tunnel was used to create a tunnel to accommodate the conventional pedicle screw.

To mitigate potential stress concentrations on the vertebrae’s processes during the pull-out experiment, a portion of the processes was sawed off. The sawing was performed perpendicular to the first tunnel and the inferior vertebral endplate (Fig 3, step 3). After the completion of these preparatory steps, the anchor was securely positioned. For the L-shaped anchor, the central pin was carefully inserted and oriented such that the lateral pin could be introduced through the second tunnel and screwed into the central pin.

### Experimental facility

**Fig 4:**
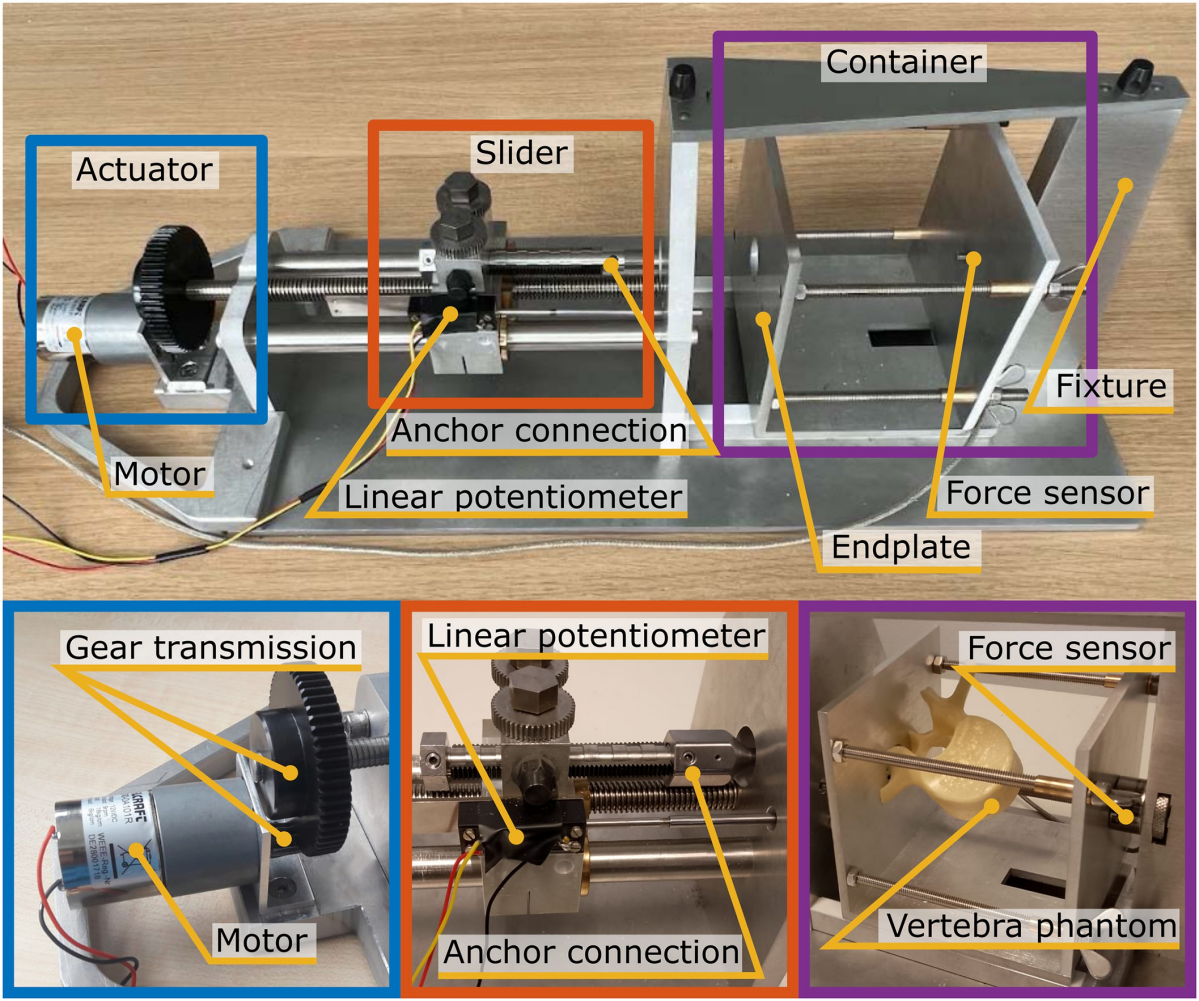
Experimental facility consisting of the actuator that pulls the slider and the anchor out of the vertebra that is contained in the container. The force is measured using the force sensor and the displacement of the L-shaped anchor with respect to the vertebra is measured using the linear potentiometer.

For the experiment a dedicated test facility was designed that is shown in Fig 4. This facility enables the controlled extraction of the anchor from the vertebra at a constant velocity while simultaneously measuring the pull-out force and relative displacement of the anchor with respect to the vertebra.

The vertebra, with the anchor securely positioned within it, was positioned within the container (Fig 4, Purple). In turn, a force sensor (Futek, LCM300, 4448 N) was connected to the container and the fixture, facilitating continuous force measurement throughout the experiment. The anchor was connected to the slider (Fig 4, Orange) through the anchor connection. The slider performs linear translations actuated by the actuation mechanism (Fig 4, Blue) consisting of an electro-motor (Modelcraft, RB 35, 1:600) with a gear transmission (9:1). The linear motion of the slider, and consequently the relative displacement between the anchor and the vertebra, was precisely measured using a linear potentiometer (Althen, 13FLP12A).

### Experimental protocol

The following steps were executed during the experiment. The vertebra, containing the securely placed L-shaped anchor or pedicle screw, was positioned within the container so that the sawing plane of the vertebra was in contact with the endplate of the container. Subsequently, the proximal end of the anchor was connected to the slider. The slider was carefully arranged to ensure contact between the vertebra and the endplate of the container. The tip of the linear potentiometer was secured to the container using an integrated magnet such that the displacement between the anchor and the vertebra could be measured.

After completing these preliminary steps, the motor was activated to initiate linear translation of the slider. This motion continued until the anchor was completely extracted from the vertebra at a constant velocity of 0.5 mm/s. Throughout this procedure, the force sensor continuously measured the pull-out force, while the linear potentiometer captured the linear displacement between the anchor and the vertebra such that the initial pull-out force could be determined. As the used linear potentiometer has a 12 mm range, the pull-out force was recorded over 12 mm.

### Data analysis

The force data were normalized by accounting for the force measured after complete pull-out of the anchor, compensating for any forces potentially exerted by the initial positioning of the slider at the start of the experiment. Furthermore, the displacement data were normalised such that the anchor starts moving at t = 0. The measured pull-out force at this point represents the initial pull-out force, as this is the force required to start the pull-out of the anchor. All data analysis was performed in Matlab R2019b.

Following the complete pull-out of the anchor, the vertebra was visually inspected for breaches in the cortical layer. Only the most severe breach was recorded. For instance, if both a breach at the pedicle and posterior breach was present, only the pedicle breach was recorded, as this type of breach holds more severe clinical implications.

## Results

**Fig 5:**
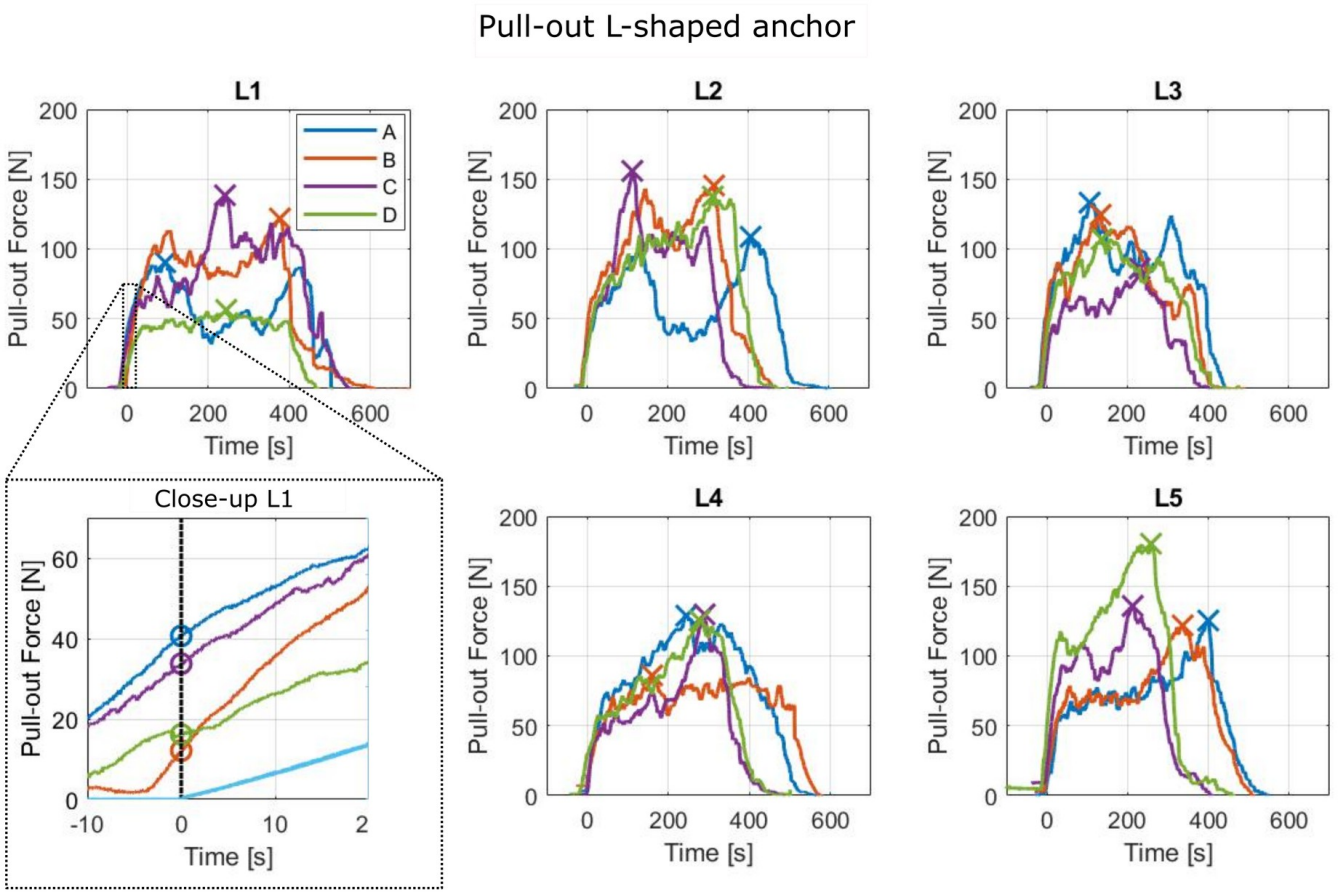
Pull-out force of the L-shaped anchor in different anchor orientations (A-D) for different vertebra (L1-L5). The maximum pull-out force is indicated with an ‘X’. The close-up shows the determination of the initial pull-out force indicated with an ‘O’.

The initial pull-out force, maximal pull-out force and damage to the vertebra after complete pull-out of the pedicle screw and the L-shaped anchor in the four evaluated orientations (A-D) is listed in Table 1 (Raw experimental data can be found in S1 Appendix). The measured pull-out force of the pedicle screw and the L-shaped anchor in the different anchor orientations (A-D) is illustrated in Fig 5. The close-up view also demonstrates how the initial pull-out force was determined.

**Table 1:** Pull-out test results for L-shaped anchor in four orientations and the pedicle screw.

Anchor	Vertebra type	Initial pull-out force [N]	Maximal pull-out force [N]	Pull-out damage
**L-shaped anchor Orientation A**	L1	41	90	Pedicle breach
	L2	34	109	Pedicle breach
	L3	53	134	Pedicle breach
	L4	10	129	Clean pull-out
	L5	7	125	Clean pull-out
**L-shaped anchor Orientation B**	L1	12	122	Pedicle breach
	L2	39	145	Pedicle breach
	L3	53	124	Pedicle breach
	L4	7	86	Clean pull-out
	L5	14	122	Clean pull-out
**L-shaped anchor Orientation C**	L1	34	138	Posterior breach
	L2	28	156	Pedicle breach
	L3	18	86	Posterior breach
	L4	3	130	Posterior breach
	L5	20	135	Pedicle breach
**L-shaped anchor Orientation D**	L1	16	56	Posterior breach
	L2	23	138	Posterior breach
	L3	37	107	Posterior breach
	L4	30	126	Pedicle breach
	L5	48	180	Pedicle breach
**Pedicle screw**	L2	203	339	Pedicle breach
	L2	40	348	Pedicle breach
	L2	3	369	Pedicle breach
	L2	142	426	Pedicle breach

**Fig 6:**
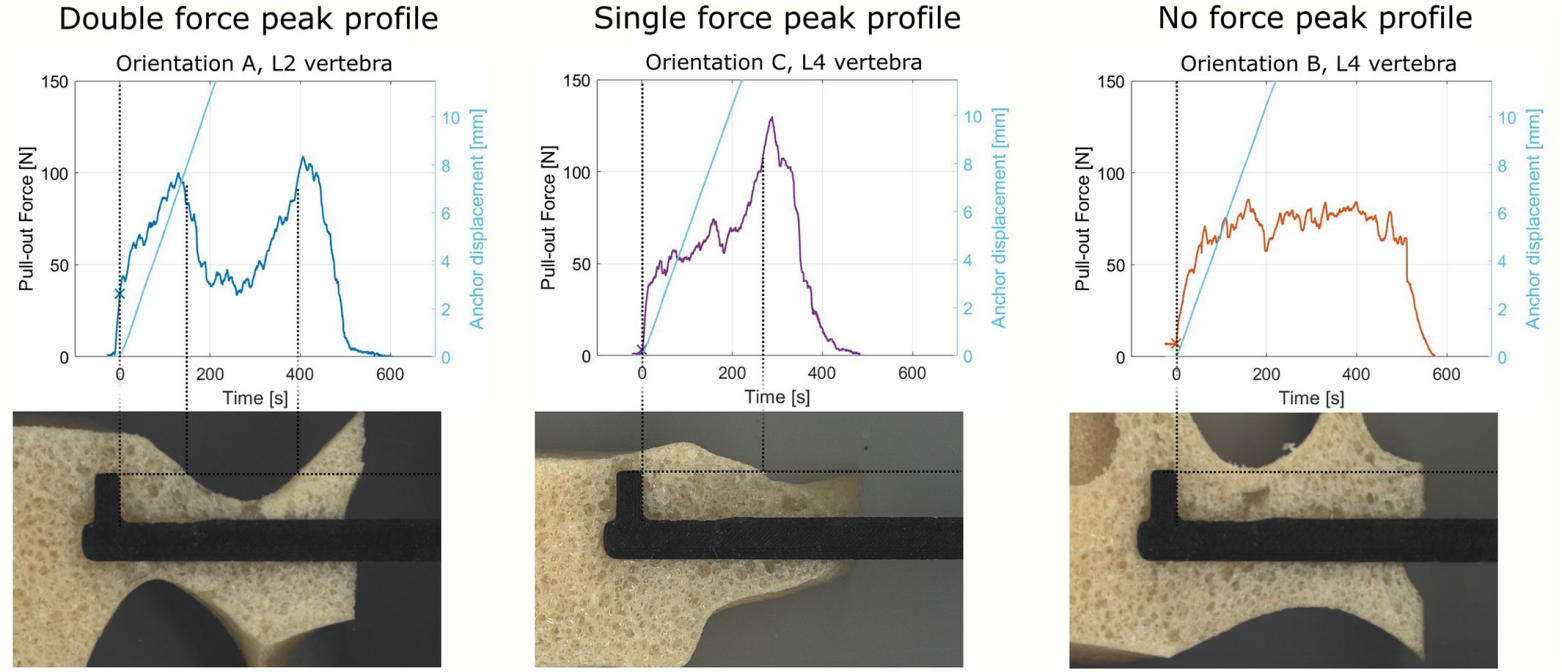
Pull-out force linked to the cross-section of the vertebra. Left: Double force peak profile linked to Orientation A in the L2 vertebra, Middle: Single force peak profile linked to Orientation C for the L4 vertebra, Right: No force peak profile linked to Orientation B in the L4 vertebra.

Based on the measured pull-out force of the L-shaped anchor, three distinct force profiles were identified: 1) a double force peak profile, 2) a single force peak profile and 3) no force peak profile. After inspecting the vertebrae cross-section, it was found that the force peaks correlate to the presence of a cortical layer, as shown in Fig 6. A double force peak profile indicates that the L-shaped anchor was pulled through two cortical bone layers, a single force peak profile indicates that the L-shaped anchor was pulled through a single cortical layer and the lack of a clear force peak indicates that the L-shaped anchor was pulled only through cancellous bone without encountering a cortical bone layer.

**Fig 7:**
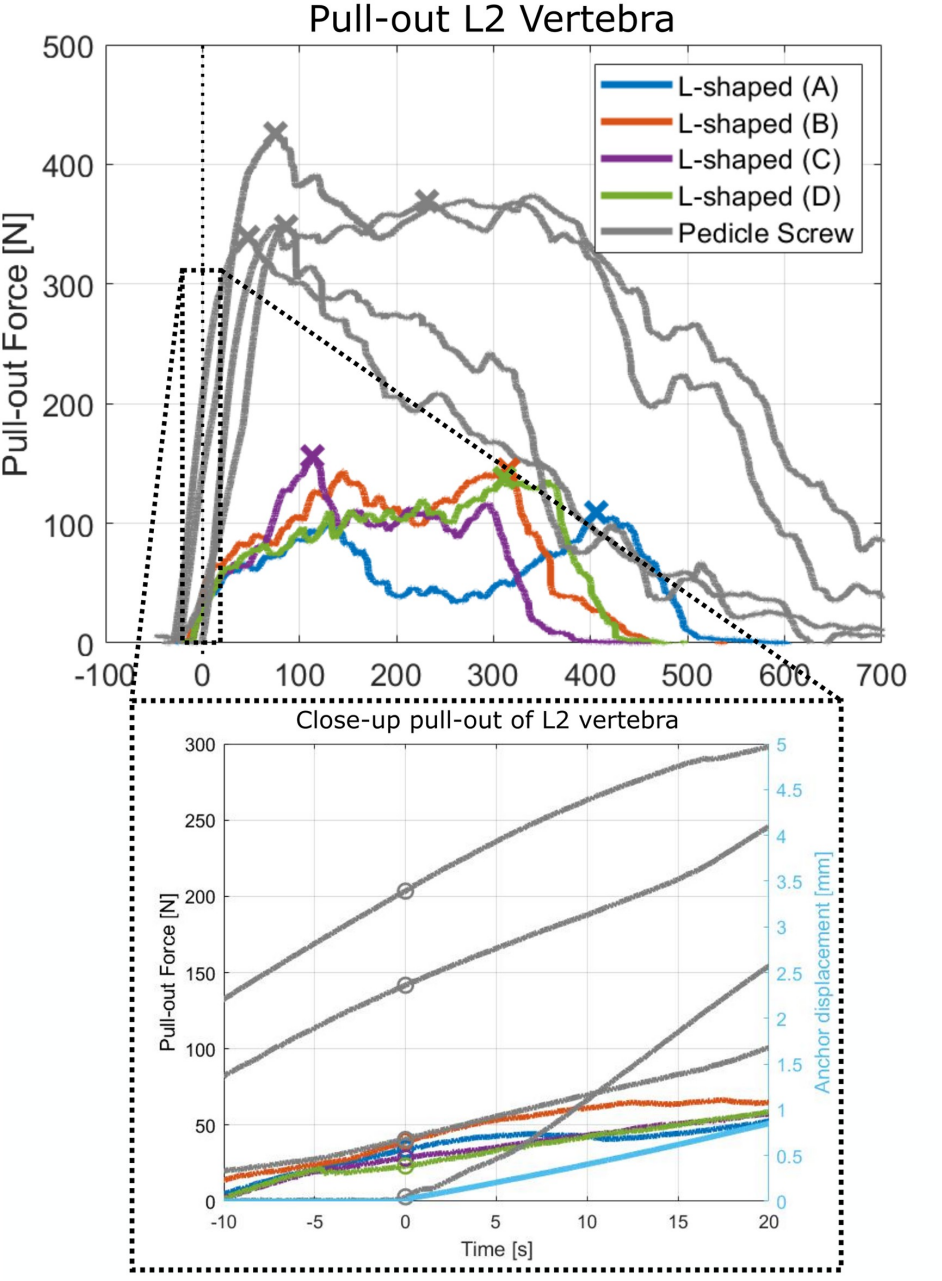
Pull-out force of the pedicle screw and L-shaped anchor in Orientation (A-D) each for four repetitions in a L2 vertebra phantom. The maximum force is indicated with an ‘X’ and the initial pull-out force is indicated with an ‘O’ in the close up.

The L-shaped anchor has a mean initial pull-out force of 23 N ± 16 N and a mean maximum pull-out force of 123 N ± 25 N. The measured pull-out force of the pedicle screw in an L2 vertebra phantom is presented in Fig 7.

**Fig 8:**
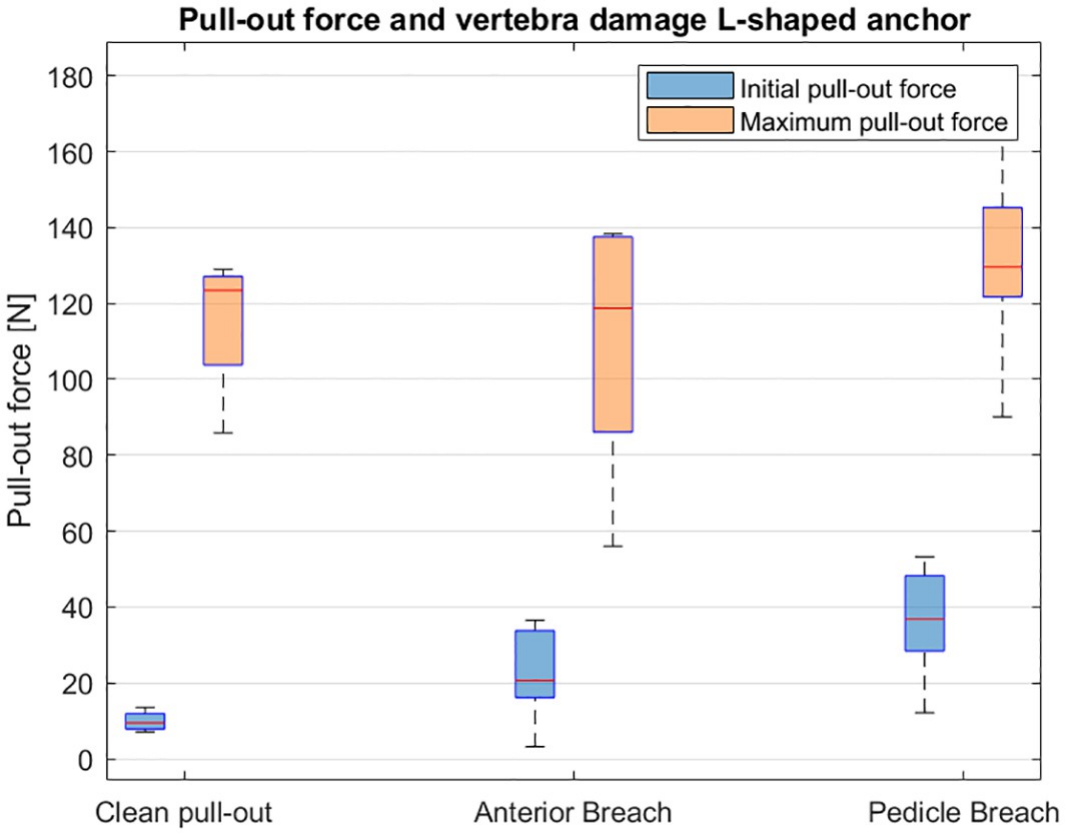
Boxplot presenting the initial (blue) and maximum pull-out force (orange) for the three identified degrees of cortical bone damage: 1) clean pull-out, 2) posterior breach and 3) pedicle breach for the experiments performed with the L-shaped anchor.

In four of the twenty pull-out experiments (20%) with the L-shaped anchor, clean pull-out (no breaches) was observed (L4A, L4B, L5A, L5B). In six cases (30%) breach of the posterior cortex was observed and in the remaining ten cases (50%) cortical breach of the pedicle was observed. Complete pull-out of the pedicle screw resulted to pedicle breach in 100% of the performed experiments. Fig 8 presents a boxplot with the initial and maximal pull-out force of the L-shaped anchor for the identified damage to the vertebra.

## Discussion

### Main findings

This study aimed to explore the effectiveness of a macro-shape lock with posterior cortex of the vertebral body to enhance the fixation strength of spinal bone anchors. An L-shaped anchor was developed and evaluated in four perpendicular orientations to assess the pull-out resistance and the safety in use. The pull-out force measurements showed three distinct force profiles: 1) double force peak profile, 2) single force peak profile and 3) no force peak profile. The pull-out force peaks could be linked to the existence of a macro-shape grip with the L-shaped anchor and the cortical bone layer indicating that a shape lock with the cortical bone layer increases the pull-out resistance of a spinal bone anchor. The mean maximum pull-out force of the L-shaped anchor was 123 N ± 25 N which is significantly lower than the maximum pull-out force of a pedicle screw (370 N ± 39 N) tested in the same bone phantoms and the pull-out force of pedicle screws reported in literature (287 N) [13]. Although the fixation strength of the L-shaped anchor is less than the conventional pedicle screw, utilizing a macro-shape lock with the proximal cortex as an add-on for the current pedicle screws could potentially increase the pull-out strength with up to 33%, although further research is required.

Alternative means to increase the fixation strength of pedicle screw reported in literature include double threaded screws or bi-cortical fixation, both have resulted in an increase fixation strength of 20% [2, 20]. These means for improved fixation can be included for the pedicle screw that is equipped with the L-shaped anchor. The use of a hydroxyapatite-coating to induce bone ingrowth can also increase the fixation strength of spinal bone anchors by 50% [21] which is more than can be expected of the L-shaped anchor. However, since the increased fixation of the hydroxyapatite-coating is established due to the surrounding bone growth into the spinal bone anchor, it takes days to weeks to establish this increased fixation, while the use of a macro-shape lock can be loaded directly. Cement augmented pedicel screws can double the pull-out resistance after placement [2]. However, removal of these screws remains a challenge without damaging the vertebra. The removal of the L-shaped anchor can be achieved as illustrated in Fig 2A. Further research is required to investigate if the use a macro-shape fixation in combination with the pedicle screw can result in the required increase in fixation strength and its ability for implant removal without damaging the vertebra.

The maximum pull-out force was found to be higher than the initial pull-out resistance for all tests with the L-shaped anchor, which suggest that the L-shaped anchor did not create a macro-shape lock in the initial position. To validate this hypothesis, a number of vertebrae were cut through such that the pull-out path of the L-shaped anchor could be investigated (Fig 6). Pull-out of the L-shaped anchor initially resulted in compression of cancellous bone. Upon contact with the cortical bone layer the maximum pull-out force was achieved resulting in the maximum pull-out force. This underscores the importance of correct initial anchor placement to establish an effective macro shape-lock with the highest pull-out resistance from the start.

**Fig 9:**
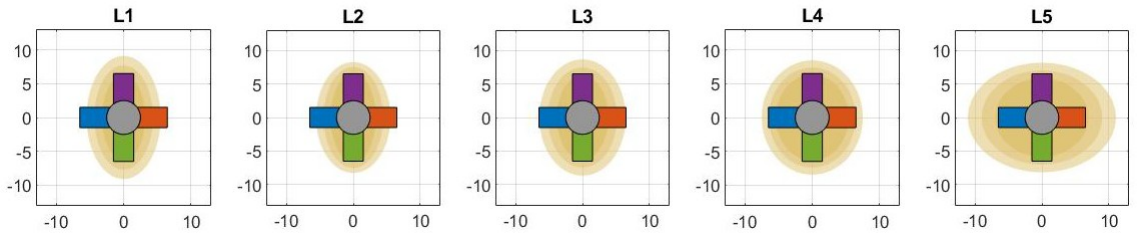
Schematic representation of the varying shape of the L1-L5 pedicle cross-section, with the L-shaped anchor in four orientations (Orientation A: blue, Orientation B: red, Orientation C: yellow, Orientation D: purple). Based om pedicle measures reported by Zindrick et al. [23].

After complete pull-out of the L-shaped bone anchor, damage to the vertebra could be observed in 80% of cases. In 50% of cases breach of the cortical layer at the pedicle could be observed, which could be an indication of damage to surrounding anatomy due to nerves and spinal cord located near the pedicle. Complete pull-out of the pedicle screw resulted in significant damage to the pedicle in 100% of the pull-out experiments with the pedicle screw. It is important to note that complete pull-out of a pedicle screw is very unlikely. Nevertheless, literature reports that in 16.2% of the anchored pedicle screws, partial pull-out of the screw is observed during rod connection in which the pedicle screw is connected to the Harrington rod [22]. A higher initial pull-out resistance of the L-shaped anchor was associated with more severe damage to the vertebra phantom after complete anchor extraction, with the degree of damage varying depending on the anchor’s orientation. A possible explanation is the variety in pedicle shape over the different vertebra as illustrated in Fig 9. Due to the ascending oval shape of the L1-L3 pedicle cross-section, orientation A and B of the L-shaped anchor are expected to create a more effective macro-shape lock with the cortical bone layer. However, the flat oval cross-section of the L5 pedicle makes orientation C and D of the L-shaped anchor more likely to generate an effective macro-shape lock. The preferred orientation of the L-shaped anchor is, therefore, dependent on the vertebra shape. Pre-operative image analysis can help in determining the most optimal anchor orientation based on the geometrical properties in the target vertebra.

Possibly, the initial pull-out resistance can be increased without increasing the risk on damage to the vertebra by initially placing the L-shaped anchor in the position in which the maximal pull-out resistance was achieved. This optimal placement of the L-shaped anchor right behind the cortical bone layer could be achieved in a safe manner by implementing real-time feedback for instance by using Diffuse Reflectance Spectroscopy (DRS) to reliably detect the cortical bone layer [24].

### Limitations and future research

The evaluation of the L-shaped anchor in this study was performed using vertebra models designed to mimic the mechanical properties of human vertebrae. Nevertheless, these models exhibited variations, such as air pockets and regions with differing structural densities, potentially influencing the measurements. Future research should incorporate *ex-vivo* and *in-vivo* experiments to provide a more accurate representation of real bone structures, helping to thoroughly assess potential fixation strength and damage to the surrounding anatomy following complete anchor pull-out.

**Fig 10:**
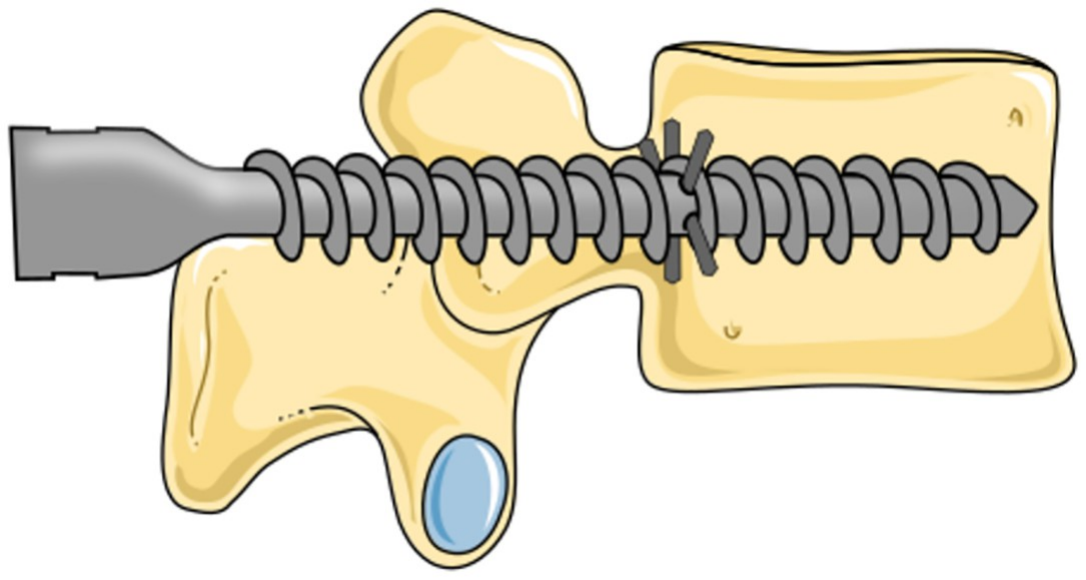
Future vision of an L-shaped anchor to increase the pull-out resistance of spinal bone anchors with the utilisation of multiple L-shaped anchors to create a larger contact area with the proximal cortex of the vertebral body which could increase the pull-out resistance further. Illustration adapted from Servier Medical Art by Servier, licensed under a Creative Commons Attribution 3.0 Unported License.

Means to enhance the fixation strength of and L-shaped add-on to the pedicle screw deserves exploration. For instance, integrating the use of multiple laterally expanding elements (Fig 10) could create an umbrella-like structure that expands behind the pedicle, establishing a macro-shape lock with the proximal cortex of the vertebral body and spreading the stress more evenly over a larger surface area. This has the potential to further enhance the anchor’s fixation strength due to increased contact with the cortical bone layer.

In this study, axial pull-out resistance of the anchor was considered. In future experiments alternative load cases such as perpendicular loads and cyclic loading can be investigated. Beyond increasing the pull-out resistance, the placement of the L-shaped anchor is thought to enhance the toggling resistance of the spinal bone anchor. Toggling, a pivoting motion of the pedicle screw around the contact point with the cortical bone layer in the pedicle, compresses the cancellous bone surrounding the screw and diminishes the screw’s fixation strength. A correctly placed L-shaped anchor creates additional contact with the cortical bone layer, preventing this toggling motion and thus increasing the anchor’s fixation strength.

## Conclusion

The L-shaped anchor presented in this study can be used to create a shape-lock with the proximal cortex of the vertebral body. The use of a macro-shape lock with the cortical bone layer represents a promising innovation in spinal instrumentation with its potential to enhance pull-out resistance with a maximum pull-out force of 123 N ± 25 N in bone phantoms. However, it is essential to acknowledge the challenges associated with this technology, including the risk of cortical breach and the technical difficulty involved in its precise placement. The presented L-shaped anchor presents the ability to increase the pull-out resistance and hold the potential to be used in combination with the current golden standard of the pedicle screw to increase the pull-out resistance. With further research and development, the use of an L-shaped anchor that utilizes a macro-shape lock with the cortical bone layer could provide a significant increase in the fixation strength of spinal bone anchors.

## Supporting information

S1 Appendix Raw experimental data. (XLSX)
